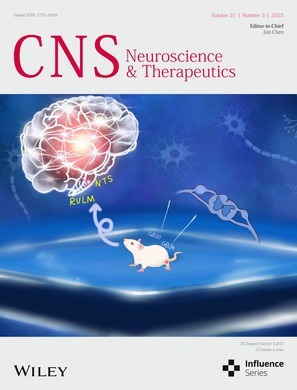# Additional Cover

**DOI:** 10.1111/cns.70388

**Published:** 2025-04-06

## Abstract

Cover image: The cover image is based on the article *Electroacupuncture Regulates Sympathetic Nerve Through the NTSGlu–RVLM Circuit to Relieve Spontaneous Pain in SNI Rats* by Wen Chen et al., https://doi.org/10.1111/cns.70327.